# Anatomic study of the vascular perfusion of the sternum and its clinical relevance in deep sternal wound infection

**DOI:** 10.3205/iprs000111

**Published:** 2017-06-06

**Authors:** Nick Spindler, Florian Kaatz, Christine Feja, Christian Etz, Friedrich-Wilhelm Mohr, Ingo Bechmann, Christoph Josten, Stefan Langer, Sabine Loeffler

**Affiliations:** 1 Department of Orthopedic Surgery, Traumatology and Plastic Surgery, University Hospital Leipzig, Leipzig, Germany; 2 Institute of Anatomy, University of Leipzig, Leipzig, Germany; 3 Department of Cardiac Surgery, Leipzig Heart Center, University of Leipzig, Leipzig, Germany

**Keywords:** deep sternal wound infection (DSWI), internal thoracic artery (ITA), vascular system of the sternum

## Abstract

**Introduction:** Deep sternal wound infections (DSWI) are a rare but devastating complication after median sternotomy. Minor perfusion in bone and soft tissue, especially after recruiting the internal mammary artery for bypass supports the development of wound infection and nonunion of the sternal bone.

The aim of the study was the macroscopic and radiological presentation of the vascular system supplying the sternum, in particular the compensating blood supply routes in the event that the internal mammary artery is no longer available after use as a bypass vessel.

**Method:** This anatomic study was carried out on the anterior chest wall of 7 specimens. The thorax plates of 7 specimens were analyzed macroscopically after microsurgical preparation. Different anatomic preparations were produced using different contrast or form-giving substances. Radiological analysis and three-dimensional reconstructions were performed to show alternative, collateral sternal vessel perfusion under estimation of the loss of the internal thoracic artery due to a bypass.

**Results:** The length of the ITA (internal thoracic artery), measured from the beginning of the first rib to the division into the superior epigastric artery and musculophrenic artery, was an average of 16.3 cm. On average, 18.5 branches were delivered from each artery, 10 medially to the sternum supply, and 8 to the intercostal muscle.

**Conclusion:** Our analysis gives an overview of the macroanatomic vessel system supplying the sternal bone, describing especially a common trunk deriving from the ITA and supplying multiple branches and playing an important role in building a collateral circulation of the sternum.

For better evaluation, in vivo CT analysis with contrast media should be performed in patients prior to the operation and directly after the use of the double ITA to demonstrate the change in perfusion of the sternum.

In the future, preconditioning of the sternum by coiling the deriving branches could become an option, although patient selection has to be improved and further analysis of the topic performed.

## Zusammenfassung

**Einleitung:** Tiefe sternale Wundheilungsstörungen sind seltene, aber verheerende Komplikationen nach medianer Sternotomie. Die verminderte Durchblutung von Knochen und Weichgewebe, vor allem nach Verwendung der Arteria thoracica interna in der kardio-chirurgischen Bypass-Chirurgie, unterstützt die Entwicklung von Wundinfektion und ein Nicht-Verheilen des sternalen Knochens. Das Ziel dieser Studie war die makroskopische und radiologische Darstellung der das Sternum versorgenden Gefäße, sowie insbesondere des kollateralen Blutkreislaufes, für den Fall, dass die Arteria thoracica interna, nach Verwendung als Bypass-Gefäß, nicht mehr zu Verfügung steht.

**Methode:** Diese anatomische Studie wurde an den Brustwandpräparaten von 7 Körperspendern durchgeführt. Das Thoraxschild dieser 7 Körperspender wurde nach mikrochirurgischer Präparation makroskopisch evaluiert und ausgewertet. Es erfolgte die Herstellung verschiedener anatomischer Präparate durch die Verwendung von entweder Kontrast- oder Form-stabilisierenden Substanzen. Radiologische Untersuchungen und dreidimensionale Rekonstruktionen wurden durchgeführt, um die alternative kollaterale Durchblutung des Sternums unter der Annahme, dass die Arteria thoracica interna als Bypass-Gefäß Verwendung fand, darzustellen.

**Ergebnisse:** Die Länge der Arteria thoracica interna (ATI), gemessen von der ersten Rippe bis zu der Aufzweigung in die Arteria epigastrica superior und die Arteria musculophrenica, betrug durchschnittlich 16,3 cm. Von jeder Arterie konnten durchschnittlich 18,5 Abgänge identifiziert werden, 10 in das Sternum ziehende und 8 in die Interkostalmuskulatur gehende Arterien.

**Fazit:** Diese Untersuchung gibt einen Überblick des makroanatomischen Gefäßsystems, welches für die Durchblutung des Sternums verantwortlich ist. Im Besonderen konnte bei den Gefäßabgängen der ATI ein sog. gemeinsamer Stamm, welcher sich im Verlauf in verschiedene Äste aufzweigt, dargestellt werden. Dieser spielt eine wichtige Rolle in der Ausbildung der kollateralen Durchblutung des Sternums.

Zur weiteren Evaluation sollten bei kardiochirurgischen Patienten, bei welchen die Bypass-Rekonstruktion mittels bilateraler Verwendung der ATI durchgeführt wird, prä- und postoperative Angio-CT-Untersuchungen durchgeführt werden. Hierdurch kann die Veränderung der sternalen Durchblutungssituation direkt sichtbar gemacht werden.

Die Präkonditionierung des Sternums durch Coiling der von der ATI abgehenden Äste und hierdurch initiierter frühzeitiger Kollateralisierung des Sternums könnte eine Verbesserung der Durchblutungssituation des sternalen Knochens erzeugen. Zuvor sollten allerdings weitere Untersuchungen zu diesem Thema durchgeführt werden.

## Introduction

With an incidence rate of 0.5–4% and mortality up to 50%, deep sternal wound infections (DSWI) are a rare but devastating complication after median sternotomy following cardiac surgery [1], [2]. 

Minor perfusion in bone and soft tissue as well as infectious maceration in the mediastinum lead to sternal bone necrosis with loosend or torn out parts of the osteosynthesis material used. It ends in persistent wound healing disorders with consecutively unstable and open thoraces. Sex, smoking, age over 74 years, diabetes mellitus as well as the use of the internal thoracic artery (ITA), especially in case of bilateral use as a bypass vessel, are the most frequent risk factors [3], [4], [5]. Better long-term survival rates, higher 10-year patency rate and a reduced risk of cardiac complications justify the more frequent utilization in comparison with the use of the saphenous vein [6]. Despite the advantages of single internal thoracic artery (SITA) and bilateral internal thoracic artery (BITA) for cardiac revascularization [7], [8], they bare a higher risk for post-operative DSWI [9], [10] due to a reduced perfusion of the bone. 

Possible sternal devascularization may lead to local bone necrosis, abscess formation and consequently, to DSWI. However, only in a few patients does the temporarily reduced blood supply have greater influence in the healing process. The reason for the regular healing process in the rest of the post-sternotomy population is the sternum’s blood supply via collateral vessels. 

The purpose of this study was to evaluate the blood supplying vessels of the sternum, show the morphometry of the ITA as well as its branches, and work out the compensating blood supply routes in the event that the internal mammary artery is no longer available after use as a bypass vessel.

## Method

This anatomic study was carried out on the anterior chest wall of 7 specimens (1 male, 6 female).

Four of the seven donor cadavers were fixed by means of an ethanol-glycerin fixative. The other three thoracic plates were harvested from “Thiel”-fixed donors. The so-called Thiel-embalmed specimens resemble in elasticity, color, and consistency the tissues of a living organism and are primarily used in surgical training [11], [12], [13]. 

Due to the special nature of Thiel’s fixation, the tissue retains its soft and elastic behavior and obliteration of the vascular system is prevented [14]. Additionally, the vascular system is preserved free of clotted blood. This allows filling materials such as latex masses (Microfil^®^) to be introduced into the vascular system. After curing, the vascular branches can be macroscopically prepared, or they can be filled with contrast media and reconstructed three-dimensionally using a CT scanner.

**Figure 1:**
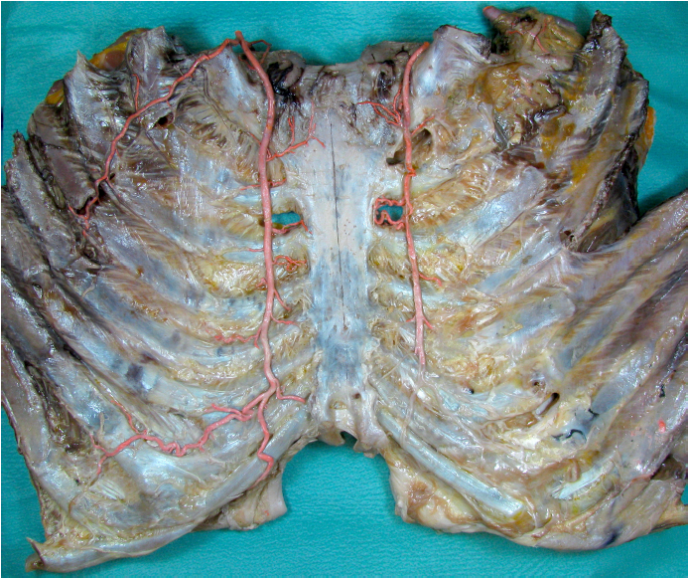
ITA and its branches

**Figure 2:**
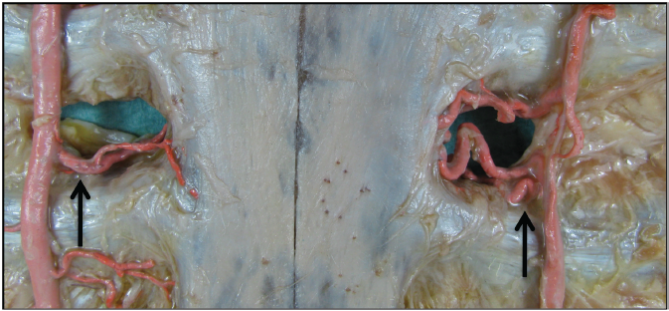
ITA and its branches of sternal and intramuscular branches (arrows: common trunk arborizing in sternal and intercostal branches)

For all donors, the thoracic plate was harvested according to the section specifications of the Institute of Anatomy of the University Hospital Leipzig. This was followed by macroscopic and microsurgical preparation of the ITA with evaluation of statistical measurements according to their anatomical architecture (length of the vessels, course as well as the individual vascular exits and perforators). The alcohol-fixed preparations were used for macroscopic and topical evaluation (Figure 1 (Fig. 1) and Figure 2 (Fig. 2))

The Thiel-fixated preparations were also macroscopically prepared according to the standardized scheme and prepared for further three tests. A thorax preparation was filled with a latex mass (Microfil^®^) and microsurgically prepared after curing. The vascular system of the second thoracic plate was filled by means of the so-called arterial mass according to Thiel, a special dextrin latex powder, which provides an excellent X-ray contrast and thus allows an angiographic representation of the vessels [11], [12]. In the third thoracic plate, the vascular system was filled with contrast media, morphologically examined with CT, and reconstructed three-dimensionally. 

The aim was to simulate the reduced supply of the sternum after bilateral use of the ITA bypass graft as the sternum is then supplied with blood. For this, the paired ITA was presented at the level of the first parasternal intercostal space. Here the two vessels were ligated. The perfusion was performed with two high-lumen catheters (18 Charrière, Virtangio^®^ Tubing Set, Fumedica, Switzerland) over the arteria femoralis dextra. After connecting to the infusion system (Virtangio^®^ Tubing Set, Fumedica, Switzerland) and attaching the pump (Virtangio^®^ Machine, Fumedica, Switzerland), the CT recording was performed with contrast medium application.

The CT images were then imported into MIMICS^®^ and segmented. MIMICS^®^, Materialise Interactive Medical Image Control System, is a special image processing program from Materialise (Leuven, Belgium), which couples 2D image files with 3D applications (Materialise 2016). For example, CT images can be segmented, three-dimensionally reconstructed using MIMICS^®^, and printed out as a model via other applications.

**Figure 3:**
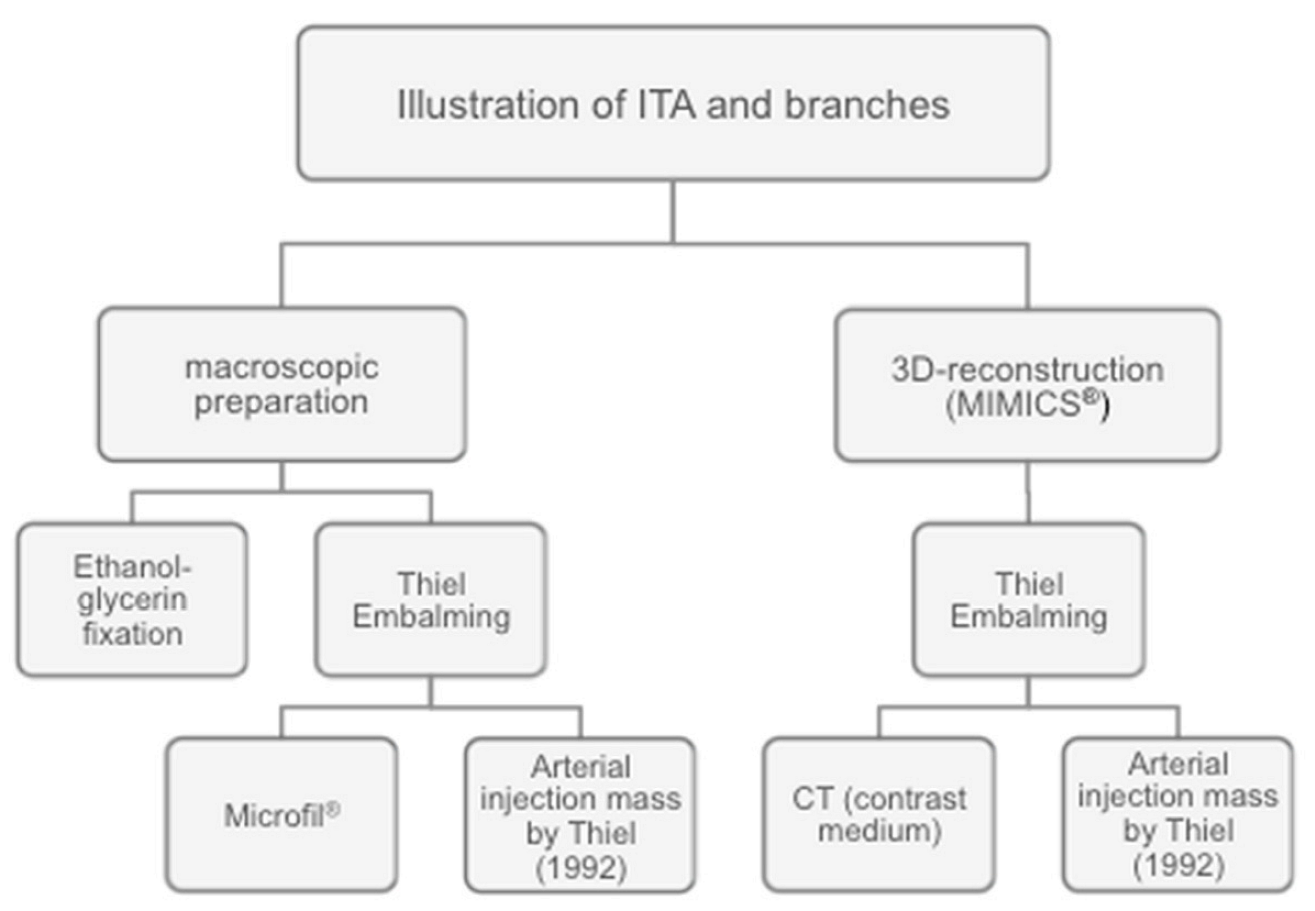
Examination algorithm

The entire examination algorithm is shown schematically in Figure 3 (Fig. 3).

## Results

**Figure 4:**
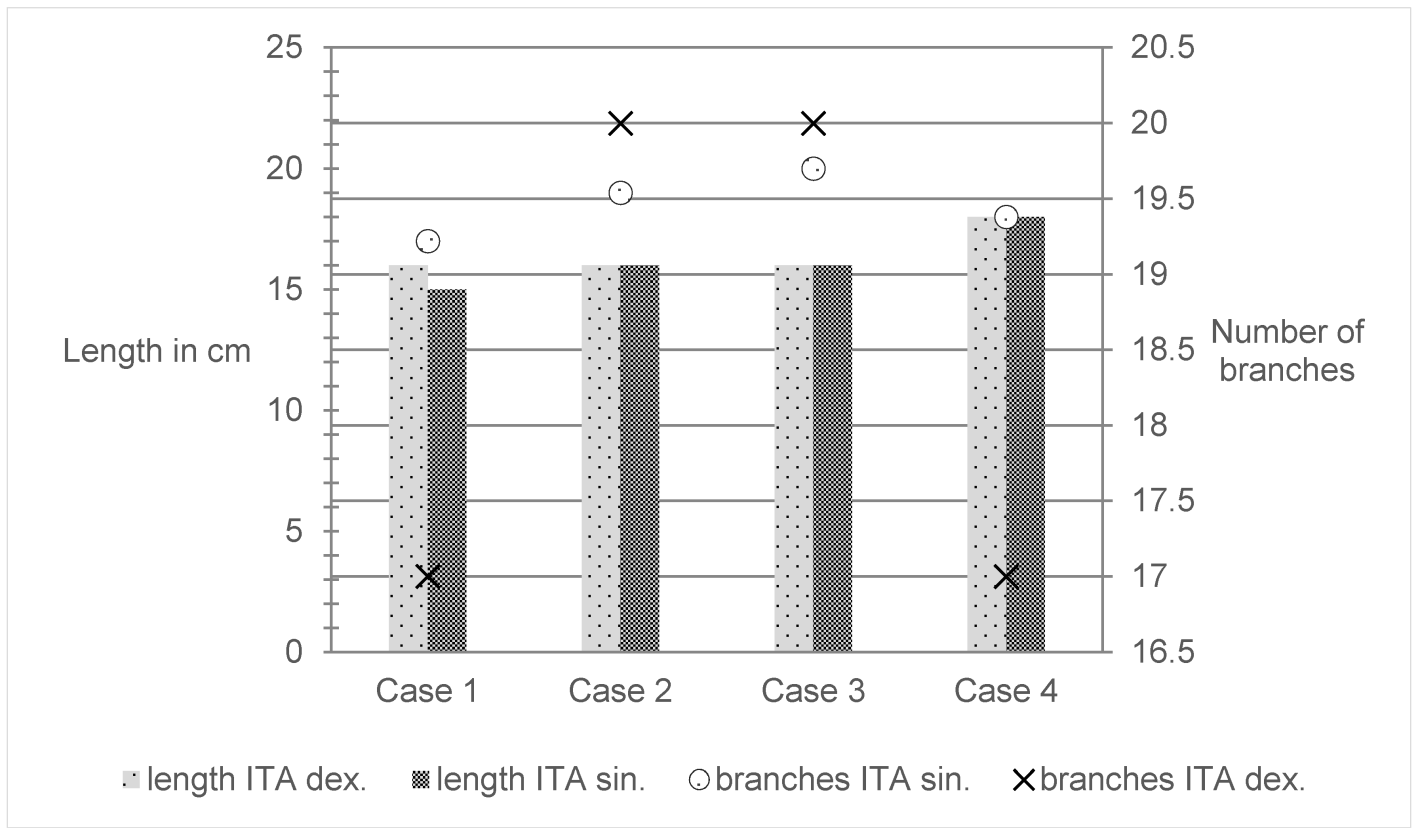
Length of each ITA and the number of branches

The aim of the macroscopic preparation was to obtain accurate information about the length, number, and region of the outgoing perforators. The test was carried out on two alcohol-fixed preparations and two preparations fixed using the Thiel method. There were no significant differences between the specimens. The length of the ITA, measured from the beginning of the first rib to the division into the superior epigastric artery and musculophrenic artery, was an average of 16.3 cm. On average, 18.5 branches were delivered from each artery, 10 medially to the sternum supply, and 8 to the intercostal muscle (Table 1 (Tab. 1) and Figure 4 (Fig. 4)).

**Figure 5:**
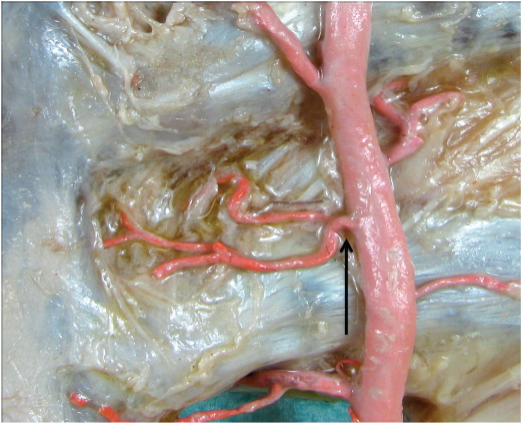
Common trunk with macroscopic formed collaterals between the sternal and the posterior intercostal arteries

The macroscopic evaluation has shown that the internal thoracic artery delivers an outlet towards the sternum in each intercostal space, as well as one in the direction of the intercostal muscle, until the artery splits at the epigastric artery and musculophrenic artery. Likewise, the course of their accompanying veins can be followed, since the blood is present after clotting in the entire vascular system. The vessels, which draw to the intercostal muscle partly formed macroscopic collaterals with the posterior intercostal arteries (Figure 5 (Fig. 5)).

After introduction of the arterial masses, the vessels were well delineated by the good X-ray properties and showed a high contrast in the CT examination. The density values were over 2900 HU. However, overlapping of other structures thus occurred, so that a sufficient differentiation between bone and vessel structure could not be shown exactly. Even in the 3D model, the structures could not be displayed separately.

**Figure 6:**
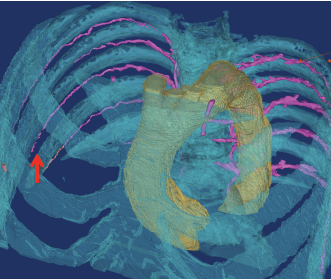
Breakdowns of the vessel structure in the CT scan

The CT morphological segmentation of the respective intercostal artery was well achieved (Figure 6 (Fig. 6)). The very low pressure with which the contrast agent was applied was a limitation. A physiological imitation of a perfusion could not be verified in this experiment. Clear breakdowns of the vessel structure were seen. Due to the non physiological imitation, this experimental setup did not permit the documentation of the sternal perfusion following simulated removal of both ITAs. 

## Discussion

Due to the fact that the ITA is the primarily used conduit for cardiac bypass surgery, fundamental knowledge about the anatomic variations of the ITA is essential to estimate the donor-site morbidity. As described by Arnold we identified four types of branches deriving from the ITA [15]. The branches proceed to the sternum, intercostally, to the mediastinum, the intercostal muscle as well its perforation and course towards the pectoralis major muscle. Also De Jesus and Acland showed that some arteries originate as proper branches with a wide diameter, which arborize in their course, while others share a common trunk anchoring up to three branches going in different directions and feeding different supplies [16]. No concise number of common trunks and the accompanying branches appear in the current literature on the subject.

Because the ITA is used primarily for bypass reasons, vessel collateralization has to compensate for the reduction of local blood perfusion of the sternum. Carrier showed that mobilization of the ITA can cause significant partial and temporary ischemia of the sternal bone, which can be more severe after use of the double ITA than single ITA. In patients with diabetes mellitus the vascularization defect was not greater. However, this temporarily lack of perfusion can be incriminated to the development of DSWI [17].

The present anatomic study created a macroscopic overview of the arterial system ensuring the blood supply of the sternum. Due to the stiff character of the specimens after fixation with alcohol, they present themselves as ideal candidates for macroscopic analysis. Using Microfil^®^ in “Thiel”-fixed body donor we enhanced the exposition of the macroscopic artery with an especially small diameter due to the low viscosity of Microfil^®^. Here we could confirm the findings of De Jesus and Acland and show that regularly four branches exist supplying the sternum, the intercostal muscle, the mediastinum and one penetrating the pectoralis major muscle. In the case that one or both of the ITA has been utilized as a bypass graft and the regular perfusion of the sternum is disturbed, the collateralization has to be established. This happens at the worst point of time, because the split sternum has to reunite itself under local hypoperfusion. The adapted forces by the applied cerclage reuniting the two halves produce local pressure and cause an additional lack of perfusion, which can potentially lead to non-union [18]. 

We handled this situation by clamping the ITA and pressure-filling the arterial system with contrast fluid while recording the findings using a three-dimensional computer tomography (CT) scan. This showed that the subcostal arteries and the muscle perforating branches are filled retrogradely. However, due to reduced pressure, we could not show an alternative perfusion of the sternum via this collateral circulation. A regular circulation pressure could not be imitated and thereby a filling of the tiny branches could not be established. A filling of the vessels by arterial mass according to Thiel was also limited by the pore size and the diameter of the passages, and could only represent the large stem vessels. By infiltration of liquid contrast medium the inflow to the sternum by the subcostal arteries and by collaterals of the A. thoraco-arcomialis could be shown using CT morphology. A complete flooding of the sternum was not observed. The reason was also the very low pressure with which the contrast agent was applied. This low pressure did not allow a complete stream of contrast of the vessels with the representation of collateral circuits in our experiments.

Carrier showed, through his MRI examinations of patients’ sternums, that after utilization of a single ITA for bypass reason, the perfusion of the sternum is reduced by approximately 13% (25% in bilateral mobilization of the ITA) [17]. The bloodstream is, as indicated by Arnold, presumably fed by interceptions of the subcostal und thoraco-acromial arteries. Discharging branches from the common trunk reach to the mediastinum, the sternum or the intercostal muscle and potentially function as a conduit for the collateral blood circulation system of the sternum [19]. 

Transferred to the clinical application it is important that the cardiac surgeon clips the vessel close to the main artery, the ITA, so that this conduit will remain intact and is able to conduct a collateral circulation to ensure the perfusion of the sternum in the first place.

DSWI are not associated to emergency interventions only but can occur after elective median sternotomy in cardiac surgery. The main risk factors are age over 74, obesity, smoking, diabetes, and male sex [3], [20]. Going through the literature we could not identify a pattern of risk factors, which will always lead to a DSWI.

Therefore it should be taken into consideration whether a preconditioning by collateralization could be initiated prior to the operation. An augmented collateral vascular system should ensure the improved perfusion of the sternum and raise the chances for bone healing. The intervention should be as non-invasive as possible. A possible attempt could be the embolization of the deriving branches of the ITA. Hereby a local hypoperfusion is created, release of growth factors is initiated and thereby, the angiogenesis is mediated [21]. Etz et al. showed a similar approach in coiling segmental arteries deriving from the aorta. Hereby a conditioning of the paraspinal vessels could be established and the secondarily performed endovascular repair showed no signs of the feared ischemic spinal cord injury [22]. Also in the attempt to precondition the perfusion of the sternum, coiling of the branches could possibly improve the vascularization of the sternal bone and hereby prevent DSWI. 

However it is very problematic to detect the ideal patients who will qualify for such an extra intervention prior to the elective operation. The known risk factors are not able to narrow the population enough.

## Conclusion

The reduced perfusion of the sternum after utilizing both ITAs for bypass is still an immanent problem after median sternotomy in cardiac surgery.

Our analysis could give an overview of the macroanatomic vessel system supplying the sternal bone, describing especially the common trunk deriving from the ITA and supplying multiple branches. This common trunk plays an important role in building a collateral circulation of the sternum.

For better evaluation, in vivo contrast CT analysis should be performed in patients prior to the operation and directly after the use of the double ITA to demonstrate the change in perfusion of the sternum.

In the future, preconditioning of the sternum by coiling the deriving branches could become an option, although patient selection has to be improved and further analysis of the topic performed.

## Notes

### Competing interests

The authors declare that they have no competing interests.

## Figures and Tables

**Table 1:**
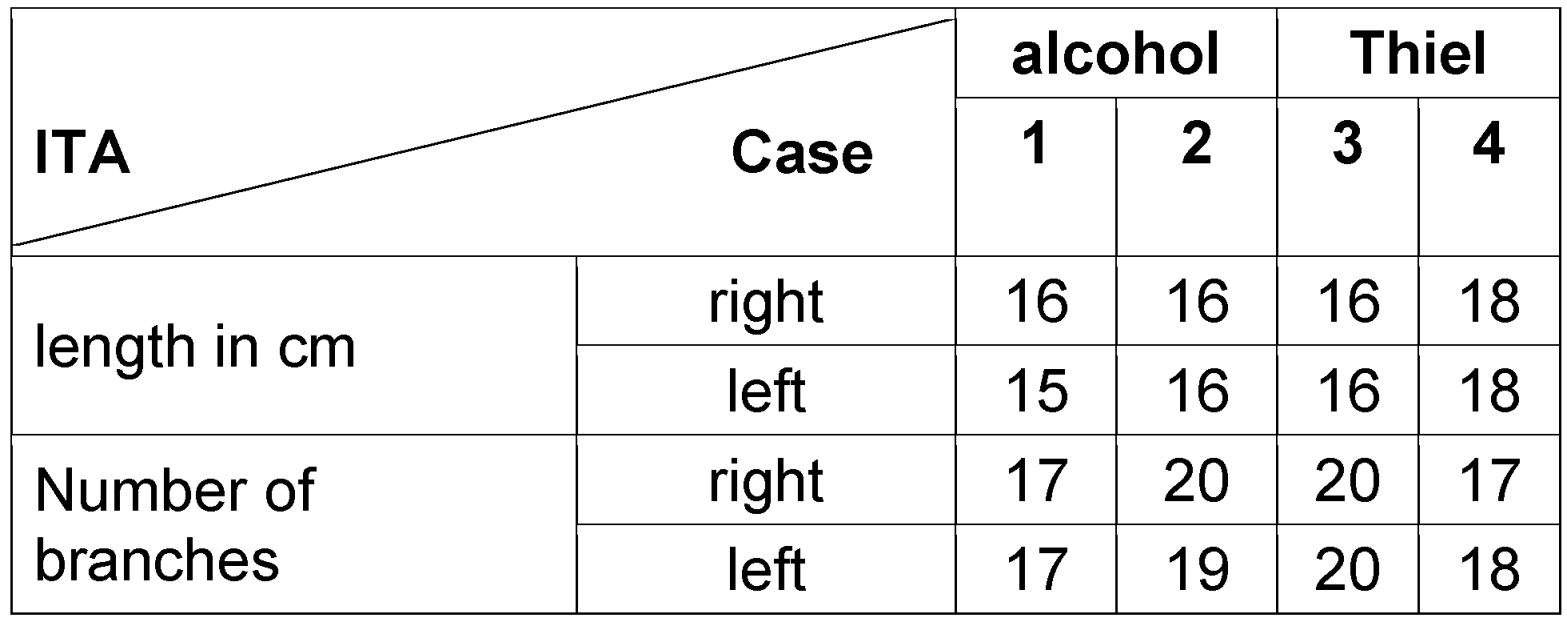
Length of each ITA
